# Mitochondrial MiRNA in Cardiovascular Function and Disease

**DOI:** 10.3390/cells8121475

**Published:** 2019-11-21

**Authors:** Rui Song, Xiang-Qun Hu, Lubo Zhang

**Affiliations:** Lawrence D. Longo, MD Center for Perinatal Biology, Department of Basic Sciences, Loma Linda University School of Medicine, Loma Linda, CA 92350, USA; xhu@llu.edu

**Keywords:** miRNA, mitomiR, mitochondria, cardiovascular disease

## Abstract

MicroRNAs (miRNAs) are small noncoding RNAs functioning as crucial post-transcriptional regulators of gene expression involved in cardiovascular development and health. Recently, mitochondrial miRNAs (mitomiRs) have been shown to modulate the translational activity of the mitochondrial genome and regulating mitochondrial protein expression and function. Although mitochondria have been verified to be essential for the development and as a therapeutic target for cardiovascular diseases, we are just beginning to understand the roles of mitomiRs in the regulation of crucial biological processes, including energy metabolism, oxidative stress, inflammation, and apoptosis. In this review, we summarize recent findings regarding how mitomiRs impact on mitochondrial gene expression and mitochondrial function, which may help us better understand the contribution of mitomiRs to both the regulation of cardiovascular function under physiological conditions and the pathogenesis of cardiovascular diseases.

## 1. Introduction

The heart pumps blood through the circulation system to provide blood supply to the body all the time. The continuous heart beating since heart formation requires ceaseless energy, which is predominantly supplied by the mitochondria in cardiomyocytes. Not surprisingly, the heart is the most metabolically active organ in the body, and the abundance of mitochondria is highest in the heart compared to other organs/tissues. Also, angiogenesis, the process of sprouting new blood vessels from preexisting vasculature, is vital for development and tissue repair/regeneration. The proliferation and migration of endothelial cells are essential for neo-vessel formation [1,2]. As expected, the proliferation and migration of endothelial cells during angiogenesis require an adequate supply of energy [3,4,5]. In addition to being the powerhouse of the cell, mitochondria also play critical roles in reactive oxygen species (ROS) generation, Ca^2+^ homeostasis, and cell death [6,7,8]. ROS derived from mitochondria could stabilize hypoxia-inducible factor 1α (HIF-1α), and HIF-1α, in turn, promotes *VEGF* expression, contributing to angiogenesis [9]. Mitochondrial dysfunction is associated with the development of many diseases, including cardiovascular diseases [8,10,11,12].

The most prominent role of mitochondria is to produce ATP through oxidative phosphorylation fulfilled by the electron transport chain (ETC) composed of four multisubunit protein complexes. More than 1000 mitochondrial proteins have been identified [13,14]. Mitochondrial proteins are of dual genetic origins. It is estimated that ~99% of mitochondrial proteins are encoded by nuclear genes and are actively imported into mitochondria through mitochondrial membrane transporters. The remaining 1% of mitochondrial proteins are encoded by the mitochondrial genome. The mitochondrion has its own protein synthesis machinery. Thirteen proteins that are components of the ETC I, III, IV, and V are encoded by mitochondrial DNA (mtDNA). Mitochondrially encoded mRNAs are translated from mtDNA on the mitochondrial ribosome (mitoribosome). Thus, appropriate regulation of mitochondrial protein synthesis is essential for normal mitochondrial function.

**Figure 1:**
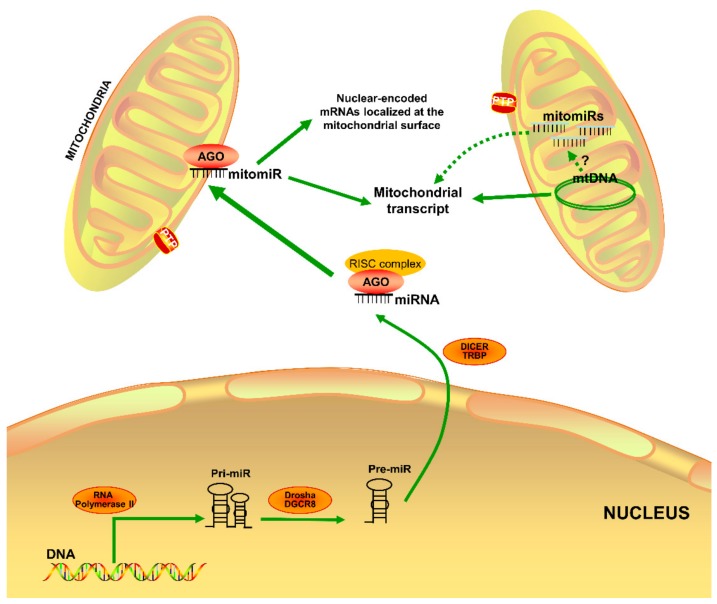
Illustration of mitomiR origin and functioning site in the cell. Some mature miRNAs are imported into mitochondria after the pre-miRNA originated from the nucleus are processed by DICER. The other mitomiRs may be originated from mitochondrial genome-derived mRNA molecules. All mitomiRs can exert post-transcriptional modification in the mitochondria. Imported miRNAs may also target and function at nuclear-encoded mRNAs localized on the mitochondrial surface. However, the mechanisms underlying mitomiRs biogenesis and action site are still poorly understood.

MiRNAs are single-stranded, noncoding RNAs with 18–23 nucleotides, participating in the regulation of gene expression at the post-transcriptional level. They mostly inhibit translation and/or induce degradation of messenger RNA (mRNA) to cause gene slicing upon complementarily binding to the 3-untranslated region (3′UTR) of the target genes [15,16,17]. MiRNAs are essential in various biological processes, including cell differentiation and proliferation, cell death, and metabolism [18,19,20,21]. MiRNA dysregulation often disrupts critical cellular processes, leading to the onset and progression of various human diseases. A given miRNA can target many target genes, and a target mRNA can also harbor binding sequences for multiple miRNAs. Not surprisingly, miRNA-based therapeutics have shown promise in treating human diseases [22,23,24,25,26,27]. Mature miRNAs are commonly present in the cytosol of cells. Intriguingly, various studies have revealed the presence of miRNAs in the mitochondrion [28,29,30]. It is not currently clear how these nuclear-encoded miRNAs are translocated into the mitochondrion. Several mitomiRs may be originated from mitochondrial genome-derived mRNA molecules. MitomiRs have been mostly found to post-translationally regulate gene expression inside the mitochondrion [31,32]. In addition, some of them may target nuclear-encoded mRNAs localized on the mitochondrial surface (Figure 1). Of importance, differentially expressed mitomiRs were observed in heart failure [33,34]. These findings implicate the important roles of mitomiRs in regulating mitochondrial gene expression and mitochondrial functions in both physiological and pathological conditions.

## 2. MitomiR Regulation of Mitochondrial Functions

### 2.1. MitomiR Biogenesis

MiRNA biosynthesis occurs in multiple enzymatic steps in both the nucleus and cytoplasm [15,35]. The transcription of miRNAs from genes takes place in the nucleus, and RNA polymerase II produces primary miRNA (pri-miRNA). The stem-loop structure in the pri-miRNA is then modified by RNAse III class of enzyme, Drosha, to form the precursor-miRNA (pre-MRA) by truncating the stem-loop. Pasha (DGCR8) helps Drosha act on the pre-miRNA to form a hairpin loop structure [36]. Exporting 5 (EXP5) and RANGTP (a GTP-binding nuclear protein) form transport machinery to export the pre-miRNA to the cytoplasm from the nucleus. EXP5 also helps protect pre-miRNA against nucleolytic degradation to reduce the number of mature miRNA in the cytoplasm while avoiding pre-miRNA from accumulating in the nucleus [37].

RNA-induced silencing complex (RISC) is formed when argonaute (Ago) binds to the Dicer generated RNA duplex along with targeted mRNA [38]. At first, Ago binds to the dsRNA to form pre-RISC before removing the passenger miRNA. The endonuclease C3PO strand facilitates to create the mature RISC, which is characterized by a strong bonding of miRNA and Ago protein [39,40]. A-form helix is the pre-arrangement of the miRNA seed sequence to facilitate efficient target scanning. Ago undergoes conformational changes made by HSC70-HSP90 protein chaperone using ATP for it to bind to the dsRNA [41]. RISC matches the 3′-UTR region to recognize the target mRNA, resulting in either the inhibition of translation or degradation of target mRNA [38].

### 2.2. MitomiRs and Mitochondrial Energy Metabolism

MitomiRs are important regulators of mitochondrial function and metabolic regulation. In-silico analysis identified mitomiRs miR-378, miR-24, miR-23b, and let-7a in liver mitochondria; these mitomiRs have been demonstrated to regulate systemic energy homeostasis, oxidative capacity, ROS generation, and mitochondrial lipid metabolism [42,43]. In addition, miR-1, miR-210, and miR-338 have been reported to enhance mitochondrial translation, regulate the mitochondrial proteome, and mitochondrial bioenergetics in myocytes [40,44,45,46]. Bioinformatics analysis showed that mitochondria enriched miR-696, miR-532, miR-690, and miR-345-3p at the early stage of the failing heart, and these miRNAs were associated with energy metabolism and oxidative stress pathways [33]. More recently, in hypoxic/reoxygenated cardiomyocytes, miR-762, miR-744, miR-92a, miR-1892, miR-150, miR-669a, miR-296-3p, miR-711, and miR-450a-3p were found to translocate into the mitochondria, whereas miR-362-5p, miR-532-5p, miR-31, miR-139-5p, miR-330, and miR-379 were decreased in the mitochondria [47]. Among them, miR-762 was demonstrated to decrease intracellular ATP levels and to increase ROS levels in cardiomyocytes [47]. Although other nuclear-encoded miRNAs may also regulate mitochondrial signaling and function [48], mitomiRs play a crucial role in post-transcriptional regulation of gene expression related to mitochondrial function (e.g., energetics and apoptosis). It is of interest to investigate the interaction of mitomiRs and other nuclear-encoded miRNAs to consummate the molecular mechanisms underlying relevant diseases in the future. Collectively, these results support a role for mitomiRs as a crucial regulator in maintaining metabolic homeostasis, which is fundamentally important to cardiovascular health.

### 2.3. MitomiRs and Mitochondrial Fission/Fusion

The mitochondrion is a very dynamic cellular organelle regularly undergoing coordinated cycles of fission and fusion (i.e., mitochondrial dynamics) [49]. Mitochondrial dynamics play important roles in alleviating and removing damaged mitochondria [50]. Mitochondrial dynamics are of critical importance in apoptosis, autophagy, inflammation, and contractile dysfunction [51,52,53]. Impaired dynamic mitochondrial behavior is frequently associated with cardiovascular diseases. MitomiRs have been shown to regulate mitochondrial fission/fusion. Forty-two mitomiRs have been reported in different cell types [54]. Of those, miR-146a, miR-34a, and miR-181a may regulate mitochondrial dynamics by targeting Bcl-2 [54]. Other mitomiRs have been demonstrated to directly target mitochondrial fission/fusion proteins [40]. MiR-484 suppressed the expression of Fis1, leading to reduced Fis1-mediated fission and apoptosis in cardiomyocytes [51]. Mitochondrial fission was also suppressed by miR-30-mediated downregulation of dynamin-related protein (Drp1) and p53 [55].

## 3. MitomiR Function in Cardiac Health and Diseases

Mitochondrial metabolism and dynamics are essential for the biological processes in physiological conditions, and alterations in either dynamics or metabolism could lead to cardiovascular disease initiation and progression [56,57]. MitomiRs regulate mitochondrial energy status, glycolysis, and the expression of genes necessary for mitochondrial metabolism to contribute to cardiovascular health and pathogenesis [33]. For example, mitomiR miR-181c played a role in electron chain complex IV remodeling in cardiomyocytes, with the levels enriched two-fold in the mitochondria compared to the whole heart [31,58,59]. MiR-181c regulates mitochondrial gene expression and affects the functioning of the mitochondria. Overexpression of miR-181c altered the levels of mRNA in the mitochondrial complex IV genes in the heart and can lead to cardiac dysfunction by regulating mitochondrial genes and reactive oxygen species (ROS) production [31,58,59]. Additionally, the downregulation of miR-181a significantly inhibited cellular apoptosis induced by H_2_O_2_ [60]. Thus, mitomiRs-mediated mitochondrial dysfunction is responsible not only for the initiation but also for the progression of cardiovascular diseases. Table 1 highlights the role of mitomiRs in cardiovascular diseases.

### 3.1. Heart Failure

Mitochondrial dysfunction is associated with the development of heart failure (HF). Mitochondria occupy nearly a third of cardiomyocyte volume and are essential for energy production, signal transduction, cell death, and ROS generation. The lack of balance between ATP demand and production is a defining feature in HF development. Various mitomiRs contribute to the altered energy metabolism, oxidative stress, cell survival, and apoptosis in HF [17,33,40]. Changes in these miRNAs influence the occurrence and progression of HF. MitomiRs such as miR-181c and miR-378 regulated particular pathways such as fatty acid metabolism, alteration in electron transport, and apoptosis under metabolic stress, and aided in mitochondrial energy metabolism [33,58]. MiR-696, miR-532, miR-690, and miR-345-3p were elevated in mitochondria of failing hearts and associated with energy metabolism and oxidative stress pathway [33]. MiR-696 was found to target PGC-1*α* to decrease both the biosynthesis of mitochondria (the mtDNA content) and fatty acid oxidation in myocytes [73]. MiR-532-3p can directly target the apoptosis repressor with the caspase recruitment domain and participate in mitochondrial fission and apoptosis in cardiomyocytes [74]. MiR-345 has been demonstrated as a cellular ATP regulator targeting genes involved in mitochondrial energy metabolism during myoblasts differentiation [75]. Other nuclear-encoded miRNAs may also regulate mitochondrial metabolism and function. For example, miR-195 induction was found to be along with decreased expression of mitochondrial deacetylase SIRT3 in failing human myocardium [76]. MiR-195 downregulated SIRT3 expression through direct 3′-UTR targeting in AC16 human cardiomyocyte-like cells, and MiR-195 KO transgenic mice exhibited reduced SIRT3 expression levels associated with hyperacetylation of key metabolic enzymes and energy depletion, leading to cardiac remodeling and heart failure [76]. It is intriguing to explore the possibility of more nuclear-encoded mitomiRs in mediating nuclear-mitochondria communication mechanisms underlying mitochondria-relevant diseases.

### 3.2. Ischemic Heart Disease

Various miRNAs regulate myocardium remodeling resulted from ischemia/reperfusion injury in ischemic heart disease [77]. Alterations in miRNA expression occur following the activation of stress signaling pathways [77,78]. It has been demonstrated that miRNAs contribute to ischemic heart disease by regulating the expression of various key mitochondrial elements in cell survival and death [40,79]. The expression of mitomiRs miR-762 and miR-210 were upregulated in myocardial infarction, while miR-1 was down-regulated [40]. MiR-1 has been demonstrated to regulate the mitochondrial electron transport chain (ETC) by targeting the mitochondrial gene cytochrome c oxidase subunit 1 (mt-COX1), and the mitochondrial gene NADH dehydrogenase subunit 1 (mt-ND1) in the ETC networks in the heart [44]. MiR-762 was recently found to largely translocate to the mitochondria and was markedly upregulated by hypoxia/reoxygenation in cardiomyocytes [47]; this directly decreased ND2 translation to decrease mitochondrial complex I enzyme activity, decrease intracellular ATP levels, increase ROS levels, and increase apoptosis in cardiomyocytes [47]. Also, knockdown of miR-762 attenuated myocardial ischemia/reperfusion injury in mice [47]. Mechanistically, we showed that enforced expression of miR-762 dramatically decreased the protein levels of endogenous ND2 but had no effect on the transcript levels of ND2. Recently, miR-210 has been reported as one of several hypoxia-induced miRNAs critical for cell survival and angiogenesis. Emerging evidence has demonstrated that the induction of miR-210-3p, a robust target of hypoxia-inducible factors, is a consistent feature of the hypoxic response in many cell types, and its overexpression has been detected in a variety of diseases with hypoxic components [80,81,82,83,84,85,86,87]. MiR-210 has been demonstrated to repress mitochondrial function by directly targeting 3′UTR of mitochondrial proteins such as mitochondrial iron-sulfur cluster homolog (ISCU), COX10, succinate dehydrogenase complex subunit D, and complex III [64,65,66]. In the heart, hypoxia-induced miR-210 has been reported to directly repress the expression of ISCU1/2 to impair mitochondrial respiration and potentially other iron-sulfur clusters dependent functions such as iron metabolism and ROS generation [88]. It should be noted that the controversial effects of miR-210 on cardiomyocytes under hypoxia conditions have been reported. Sun et al. and our previous study demonstrated that miR-210 induced oxidative stress, inhibited mitochondrial function, and promoted cell death in fetal cardiomyocytes [89,90]. However, Mutharasan et al. showed that overexpression of miR-210 reduced fetal cardiomyocyte death in response to oxidative stress and reduced ROS production through Akt- and p53-dependent pathways [91]. Furthermore, overexpression of miR-210 reduced cell death and improved cardiac function and angiogenesis after acute myocardial infarction in vivo [84]. Collectively, the exact role of miR-210 targeting mitochondria in ischemic heart disease remains unclear. Given the crucial role of mitochondria in ischemic heart diseases, it is important to further investigate the role of miR-210 targeting mitochondria in other cell types such as fibroblasts, inflammatory cells, and endothelial cells in the heart.

Other nuclear-encoded miRNAs have been found to directly regulate mitochondrial proteins in the ischemic heart. For example, the upregulation of miR-15/16 family and miR-195 suppressed ATP levels and induced mitochondrial fusion, by acting on their common target ADP-ribosylation factor-like 2 to influence cardiomyocyte survival and contribute to myocardial infarction [61,62,63]. Inhibition of miR-15 family protected against ischemia/reperfusion heart injury in vivo through directly de-repression of pyruvate dehydrogenase kinase 4 and serum/glucocorticoid-regulated kinase 1, which regulate mitochondrial function and apoptosis, respectively [61]. In contrast, among miRNAs that were upregulated after cardiac stress, miR-499 and miR-214 had a protective function. MiR-499 downregulated calcineurin and Drp-1, both involved in mitochondrial fission, to influence cardiomyocyte apoptosis [53]. The upregulation of miR-499 reduced infarct size and apoptosis, while its antagonization had an opposite effect [53]. Myocardial infarction also led to an increase in miR-214, which boosted protection during ischemia by reducing calcium overload and promoting cardiomyocyte survival, at least partly by inhibiting mitochondrial signaling, including cyclophilin D, a regulator of the mitochondrial permeability transition pore and pro-apoptotic Bcl-2-like protein 11 [92]. Nuclear-encoded mitochondrial miRNAs may localize on the surface of the mitochondrion, and this localization could enhance their translation. Therefore, three aspects for identification of mitomiRs need further investigation: (1) to validate miRNAs translocating to mitochondria; (2) to determine what nuclear-encoded mitochondrial miRNAs localize on the mitochondrial surface; (3) to identify miRNAs that target the mRNAs on the mitochondrial surface.

### 3.3. Cardiac Hypertrophy

Redox homeostasis and mitochondrial dynamics control cardiac remodeling and cardiac hypertrophy. MiRNAs targeting mitochondrial function and morphology regulation have been revealed in this remodeling process. MitomiR-146a was found to be upregulated in the heart in both mouse models of pressure overload and in patients who have aortic stenosis [67,93]. Mitochondrial protein dihydrolipoamide succinyltransferase (DLST) is a potential target of miR-146a and functions as a tricarboxylic acid [67]. AntimiR-146a treatment during pressure overload can give beneficial effects by de-repressing DLST to create a favorable metabolic profile with preserved both glucose oxidation and fatty acid oxidation in cardiomyocytes, which leads to diminished hypertrophy and an improvement in cardiac function [67]. Recently, miR-1 was found to effectively bind to the mitochondrial calcium uniporter (MCU) mRNA to influence mitochondrial Ca^2+^ flux, contributing to cardiac hypertrophy [48]. In addition, miR-30 has been reported to antagonize apoptosis of cardiac cells through negatively regulating Drp1, an initiator of mitochondrial fission, Bcl-2, and Bnip3L/Nix, leading to apoptosis [55,68]. MiR-485-5p directly downregulated mitochondrial anchored protein ligase, an important contributor in the mitochondrial fission process, and upregulated mitochondrial fusion protein 2 (Mfn2) in primary hypertrophic cardiomyocytes [69]. In vivo, miR-485-5p agomir suppressed cardiac hypertrophy in mice [69]. Thus, mitomiRs may be potential markers in cardiac hypertrophy and may negatively or positively regulate the progression of pathological cardiac remodeling through modulation of mitochondrial fusion-fission and function.

### 3.4. Diabetic Heart

Diabetes mellitus greatly increases the risk of and mortality from heart disease. Diabetic heart diseases include coronary heart disease, heart failure, and diabetic cardiomyopathy. The diabetic heart is characterized by insulin resistance, reduced cellular glucose uptake and oxidation, and increased mitochondrial fatty acid uptake and oxidative stress, mitochondrial dysfunction, and cardiomyocyte apoptosis [94,95]. Recent studies revealed the aberrant expression of mitomiRs contributing to the pathogenetic processes of diabetic heart diseases [71,72]. In the diabetic condition, ATP synthase activity was shown to decrease, correlating with increased mitochondrial miRNA-378 in the heart. Mitochondrial miRNA-378 acts as a potential target for reinstating cardiac mitochondrial bioenergetic function and, consequently, cardiac pump function [70,71]. More recently, in type 2 diabetic heart, miR-92a was found to be downregulated in cardiac mitochondria. MiR-92a can translocate into mitochondria to counter mitochondrial gene cytochrome-b downregulation. Overexpression of miR-92a enhanced mitochondrial translation and reduced ROS production and lipid deposition, leading to improving diabetic cardiomyopathy [72].

Taken together, deregulated mitomiRs are potentially involved in the etiology and pathogenetic processes of cardiac diseases. An in-depth understanding of the functional roles of mitomiRs and molecular mechanisms, including their interaction with other nuclear-encoded miRNAs in the pathogenesis of diabetic heart, remains to be further explored.

## 4. MitomiR Regulation of Angiogenesis

**Figure 2:**
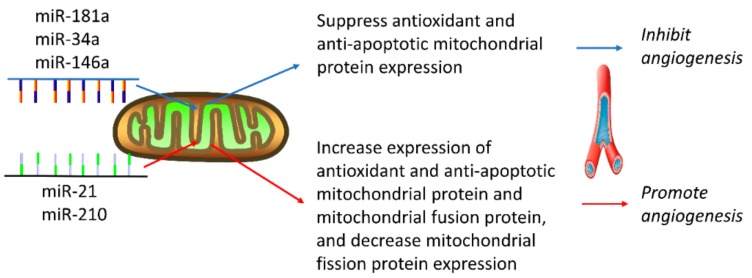
MitomiRs activity in vascular diseases: anti- or pro-angiogenic role. Some mitomiRs, such as miR-181a, miR-34a, and miR-146a, inhibit angiogenesis through suppressing antioxidant and anti-apoptotic mitochondrial protein to increase ROS production and cell apoptosis. Meanwhile, others, such as miR-21 and miR-210, play a pro-angiogenic role through enhancing mitochondria-mediated apoptosis pathways.

Angiogenesis refers to the formation of new blood vessels from preexisting ones. The process is regulated by angiogenic factors and involves cell proliferation, tube formation, migration, differentiation [96]. Angiogenesis plays an important role in physiological and pathological processes such as aortic dissection, wound healing, the formation of granulation tissues, deep venous thrombosis, stroke, atherosclerosis, tumor, and other angiogenic disorders [97]. As such, angiogenic regulation becomes an essential therapeutic strategy for cancer and vascular diseases. Although less than 10% of the 400+ miRNAs identified in the human genome are involved in angiogenesis, miRNAs play significant roles in angiogenesis [97]. Although the role of mitomiRs is still largely unknown, some studies suggest that mitomiRs play a crucial role in angiogenesis via regulating mitochondrial function and energy metabolism (Figure 2).

### 4.1. MiRNAs that Inhibit Angiogenesis

Several studies have documented that some mitomiRs promote mitochondrial dysfunction via Bcl-2 downregulation [98,99]. Bcl-2 is an antioxidant and antiapoptotic mitochondrial protein and regulates mitochondrial fission/fusion [100,101]. The mitomiR-induced Bcl-2 deregulation may lead to a state of dysfunctional mitochondria, increased oxidative stress, chronic low-grade inflammation, and increased apoptosis rates in angiogenesis-related diseases. Giuliani et al. found that mitomiR-181a, -34a, and -146a, were upregulated and localized to mitochondria and downregulated Bcl-2 in human aging endothelial cells [98]. Further, overexpression of these mitomiRs was found to decrease Bcl-2 expression, leading to mitochondrial permeability transition pore opening, activation of caspase-1 and 3, and cell apoptosis [98]. MitomiRs has been demonstrated to inhibit angiogenesis and vascularization [102]; however, the molecular mechanisms involved in mitomiRs-inhibited angiogenesis, with particular emphasis on those associated with cardiovascular disease, need further investigation.

### 4.2. MiRNAs that Promote Angiogenesis

The stimulation of angiogenesis is a characteristic feature of hypoxia [103]. Hypoxia induces both miR-21 and miR-210 [104]. In vivo inhibition of either miR-21 or miR-210 attenuated hypoxic vasoconstriction and subsequent vascular remodeling [105]. Anti-miR-21 treatment downregulated the anti-apoptotic mitochondrial membrane protein Bcl2, which blocked apoptotic cell death and promotes tumor angiogenesis [106]. It also promotes the tube forming capacity of primary bovine retinal microvascular endothelial cells [97]. Hypoxia-induced miR-210 was found in vascular cell types, including murine and human pulmonary arterial endothelial cells, human aortic endothelial cells, and human pulmonary arterial smooth muscle cells [88]. Hypoxia-induced miR-210 directly repressed the expression of ISCU1/2, leading to impairing mitochondrial respiration and metabolism in vascular cells [88]. Recently, it has been shown that overexpression of miR-210 in endothelial progenitor cells increases MMP and ATP levels, as well as decreases mitochondrion fragmentation through reducing Drp1 expression and increasing Mfn2 expression, which reduces hypoxia/reoxygenation-induced endothelial cell apoptosis, ROS overproduction, and angiogenic dysfunction [107]. Yet, the roles and mechanisms of mitomiRs in targeting mitochondria and regulating mitochondrial function in angiogenesis-associated cardiovascular disease remain largely unknown and await further investigation.

## 5. Conclusions

Cardiovascular disease is the leading cause of morbidity and mortality worldwide. Despite extensive studies on the pathogenesis, the underlying pathophysiological mechanisms are still not fully understood. Not surprisingly, research of cardiovascular disease remains a very active field. Accumulating evidence implicates aberrantly expressed miRNAs in human diseases, including cardiovascular diseases. Recent studies have revealed the presence of miRNAs in the mitochondrion and the regulation of mitochondrial function by these mitomiRs in both physiological and pathological conditions of the cardiovascular system. These findings open a new field to explore novel molecular mechanisms controlling mitochondrial gene expression in cardiovascular disease. Understanding molecular mechanisms underlying mitomiRs modulation of cardiac dysfunction and angiogenesis will facilitate developing effective therapeutic approaches for the management of cardiovascular diseases.

## Figures and Tables

**Table 1:** The role of mitomiRs in cardiac diseases.

miRNA	Cell Type/Tissue	Targeting Mitochondria	Pathology	Reference
miR-181c and miR-378	Human and rat heart	fatty acid metabolism, electron transport activity, and energy metabolism pathways	Heart failure	[33,58]
miR-696, miR-532, miR-690, miR-345-3p	Human heart	fatty acid biosynthesis, energy metabolism, and oxidative stress pathways	Heart failure	[33]
miR-762	Neonatal rat cardiac myocytes, pig and mouse heart	energy metabolism pathways (pyruvate dehydrogenase kinase 4, serum/glucocorticoid-regulated kinase 1), and mitochondrial fusion regulators	Ischemic heart disease	[61,62,63]
miR-210	Fetal rat cardiomyocytes, and mouse embryonic fibroblasts	energy metabolism and oxidative stress pathways (mitochondrial iron-sulfur cluster homologue (ISCU1/2))	Ischemic heart disease	[64,65,66]
miR-146a	Human and mouse heart, and neonatal rat cardiomyocytes	energy metabolism and oxidative stress pathways (dihydrolipoamide succinyltransferase (DLST)	Cardiac hypertrophy	[67]
miR-1	Mouse heart	mitochondrial calcium signaling (the mitochondrial calcium uniporter (MCU))	Cardiac hypertrophy	[64,65,66]
miR-30	Neonatal rat cardiac cells and rat heart	mitochondrial apoptosis signaling (Bcl-2 and Bnip3L/Nix) and the mitochondrial fission regulator dynamin-related protein 1 (Drp1)	Cardiac hypertroph	[55,68]
miR-485-5p	Neonatal rat cardiomyocytes and mouse heart	mitochondrial fusion-fission regulators (mitochondrial anchored protein ligase and mitochondrial fusion protein2 (Mfn2))	Cardiac hypertrophy	[69]
miRNA-378	Mouse heart and HL-1 cardiomyocyte	ATP synthase membrane subunit 6	Diabetic heart	[70,71]
miR-92a	Neonatal rat cardiac myocytes, and mouse heart	mitochondrial gene cytochrome-b	Diabetic heart	[72]
